# Phylogeography and evolutionary history of hepatitis B virus genotype F in Brazil

**DOI:** 10.1186/1743-422X-10-236

**Published:** 2013-07-16

**Authors:** Francisco CA Mello, Oscar C Araujo, Barbara V Lago, Ana Rita C Motta-Castro, Marcia Terezinha B Moraes, Selma A Gomes, Gonzalo Bello, Natalia M Araujo

**Affiliations:** 1 Laboratory of Molecular Virology, Oswaldo Cruz Institute, FIOCRUZ, Rio de Janeiro, RJ, Brazil; 2 Department of Biochemistry and Pharmacy, Federal University of Mato Grosso do Sul, Campo Grande, MS, Brazil; 3 Laboratory of AIDS and Molecular Immunology, Oswaldo Cruz Institute, FIOCRUZ, Rio de Janeiro, RJ, Brazil

**Keywords:** Hepatitis B virus, Genotype F, Bayesian framework, Phylogeography, Brazil

## Abstract

**Background:**

Hepatitis B virus (HBV) genotype F (HBV/F) is considered to be indigenous to the Americas, but its emergence and spread in the continent remain unknown. Previously, only two HBV/F complete genome sequences from Brazil were available, limiting the contribution of Brazilian isolates to the phylogenetic studies of HBV/F. The present study was carried out to assess the proportion and geographic distributions of HBV/F subgenotypes in Brazil, to determine the full-length genomic sequences of HBV/F isolates from different Brazilian geographic regions, and to investigate the detailed evolutionary history and phylogeography of HBV/F in Brazil.

**Methods:**

Complete HBV/F genomes isolated from 12 Brazilian patients, representing the HBV/F subgenotypes circulating in Brazil, were sequenced and analyzed together with sequences retrieved from GenBank, using the Bayesian coalescent and phylogeographic framework.

**Results:**

Phylogenetic analysis using all Brazilian HBV/F S-gene sequences available in GenBank showed that HBV/F2a is found at higher frequencies countrywide and corresponds to all sequences isolated in the Brazilian Amazon Basin. In addition, the evolutionary analysis using complete genome sequences estimated an older median ancestral age for the Brazilian HBV/F2a compared to the Brazilian HBV/F1b and HBV/F4 subgenotypes, suggesting that HBV/F2a represents the original native HBV of Brazil. The phylogeographic patterns suggested a north-to-south flow of HBV/F2a from Venezuela to Brazil, whereas HBV/F1b and HBV/F4 strains appeared to have spread from Argentina to Brazil.

**Conclusions:**

This study suggests a plausible route of introduction of HBV/F subgenotypes in Brazil and demonstrates the usefulness of recently developed computational tools for investigating the evolutionary history of HBV.

## Background

Hepatitis B virus (HBV) infection is a major public health problem worldwide. At least two billion people (one-third of the global population) have been infected at some point during their lives, and more than 240 million suffer from chronic HBV infection, putting them at an increased risk for liver cirrhosis and hepatocellular carcinoma [1].

The HBV genome is a partially double-stranded DNA molecule of approximately 3.2 kilobases in length. It has a highly compact coding structure consisting of four overlapping reading frames, which are designated P (polymerase), S (surface), C (core) and X (HBx protein). HBV is a DNA virus that employs the error-prone polymerase reverse transcriptase as part of its replication process [2]. For this reason, most estimates of the nucleotide substitution rate for HBV range between 10^-4^ to 10^−6^ substitutions per site per year (s/s/y) [3-8], which is far higher than those observed in other dsDNA viruses.

Eight HBV genotypes (A-H) have been recognized based on a sequence divergence of more than 8% throughout the genome [9-11]. More recently, two additional genotypes (I and J) were tentatively proposed [12-14]. There is a great deal of diversity within genotypes, leading to the division of some genotypes into subgenotypes [15,16]. The evolution of HBV is strikingly highlighted by the geographical distribution of the genotypes: Genotypes A (HBV/A) and HBV/D have worldwide distributions; HBV/B and HBV/C are found essentially in Asia; HBV/E is confined to West and Central Africa; HBV/G has been found in Europe, the USA and Japan (and may be ubiquitous); and HBV/H is found in Central America and the southern part of the USA [10,17].

The evolutionary history of HBV/F, which is one of the most divergent HBV genotypes [18], is not well understood due to the relative lack of appropriate studies. It is closely related to HBV/H [9], and probably originated in Amerindian populations, as it has been found in the native populations of Alaska, Central America and South America [9,19-25]. HBV/F has been divided into four genetically distinct subgenotypes (HBV/F1-F4), two of which have been further subdivided. HBV/F1 is found in Central America (HBV/F1a) and Alaska and South America (HBV/F1b). HBV/F3 is found in Central America and in northern South America, whereas HBV/F2 (HBV/F2a and HBV/F2b) [20] and HBV/F4 are found in South America [26].

Brazil is the largest country in the Southern Hemisphere, corresponding to almost half of the area of South America, and is divided broadly into five geographic regions: north, northeast, central west, southeast and south. Brazil has a highly miscegenated population, with HBV/A, HBV/D and HBV/F circulating among Brazilian HBV carriers [23,27-29]. A large-scale study on the geographic distribution of HBV genotypes in Brazil previously showed a low countrywide prevalence of HBV/F (13%) [23]; this differs from other Latin American countries, where HBV/F prevails [20,30-32]. However, a few studies on isolated indigenous communities of Brazil have shown a predominance of HBV/F [23,33,34]. Additionally, the Brazilian Amazon region is a highly endemic area for HBV and hepatitis delta virus (HDV); in this region, HBV/F has a higher frequency among HBV-HDV co-infected patients than in the general HBV-monoinfected population [35,36]. Previously, only two Brazilian HBV/F complete genome sequences, GenBank accession numbers X69798 [37] and HE981181 [38] were available, limiting the contribution of Brazilian isolates to the phylogenetic studies of HBV/F.

Here, we examined the proportion and geographic distributions of HBV/F subgenotypes in Brazil, determined the full-length genomic sequences of 12 HBV/F isolates from different Brazilian geographic regions, and used the Bayesian coalescent and phylogeographic framework to investigate the origin and spread of HBV/F subgenotypes in Brazil.

## Results

### Phylogenetic analysis of HBV/F S-gene sequences from Brazil

**Figure 1:**
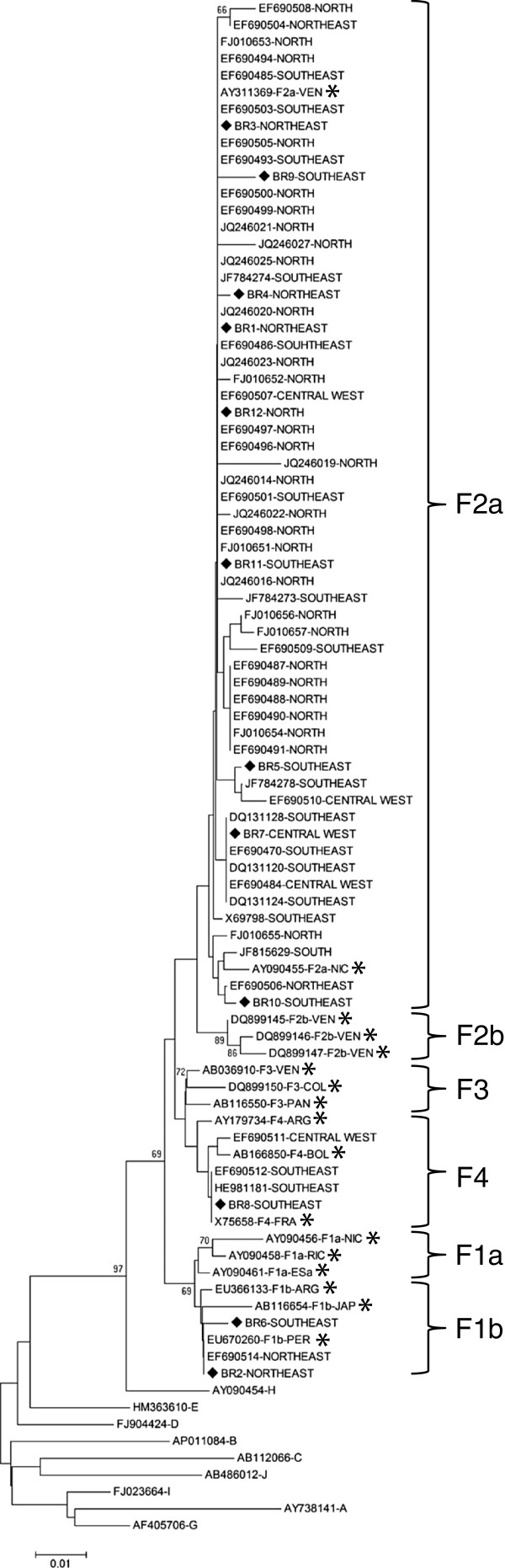
**Phylogenetic tree of partial S-gene (403 bp) sequences inferred by using the neighbor-joining method.** Values at internal nodes indicate percentage of 1000 bootstrap replicates that support the group. The horizontal bar indicates the number of nucleotide substitutions per site. Brazilian sequences available in GenBank are designated by their accession numbers, followed by the geographic region of origin. The sequences generated in this study are denoted BR, followed by the sample number (1 to 12) and the geographic region of origin, and are identified with ♦. HBV/F reference sequences are indicated by their accession numbers, followed by the respective genotype/subgenotype and the abbreviation of the origin country (ARG: Argentina; BOL: Bolivia; COL: Colombia; ESa: El Salvador; FRA: France; JAP: Japan; NIC: Nicaragua; PAN: Panama; PER: Peru; RIC: Costa Rica; VEN: Venezuela), and are identified with *. Reference sequences for the other genotypes are indicated by their accession numbers, followed by the HBV genotype.

To identify the HBV/F subgenotypes circulating in Brazil and examine their proportion and geographic distributions, we initially performed a phylogenetic analysis using all Brazilian HBV/F S-gene sequences available in GenBank (n=53) (Figure 1). We focused on S-gene sequences because only two full-length Brazilian HBV/F genomes were available prior to the present study. Of these 53 Brazilian HBV/F S-gene sequences, 29 (54.7%) are from the north, 16 (30.2%) from the southeast, 4 (7.5%) from the central west, 3 (5.7%) from the northeast, and 1 (1.9%) from the south of Brazil.

The results of our phylogenetic analysis revealed that HBV/F2a is the most prevalent HBV/F subgenotype in Brazil, with 92.5% of the sequences clustering together with HBV/F2a reference sequences. HBV/F4 (5.7%) and HBV/F1b (1.9%) were also found among the Brazilian sequences (Figure 1).

**Figure 2:**
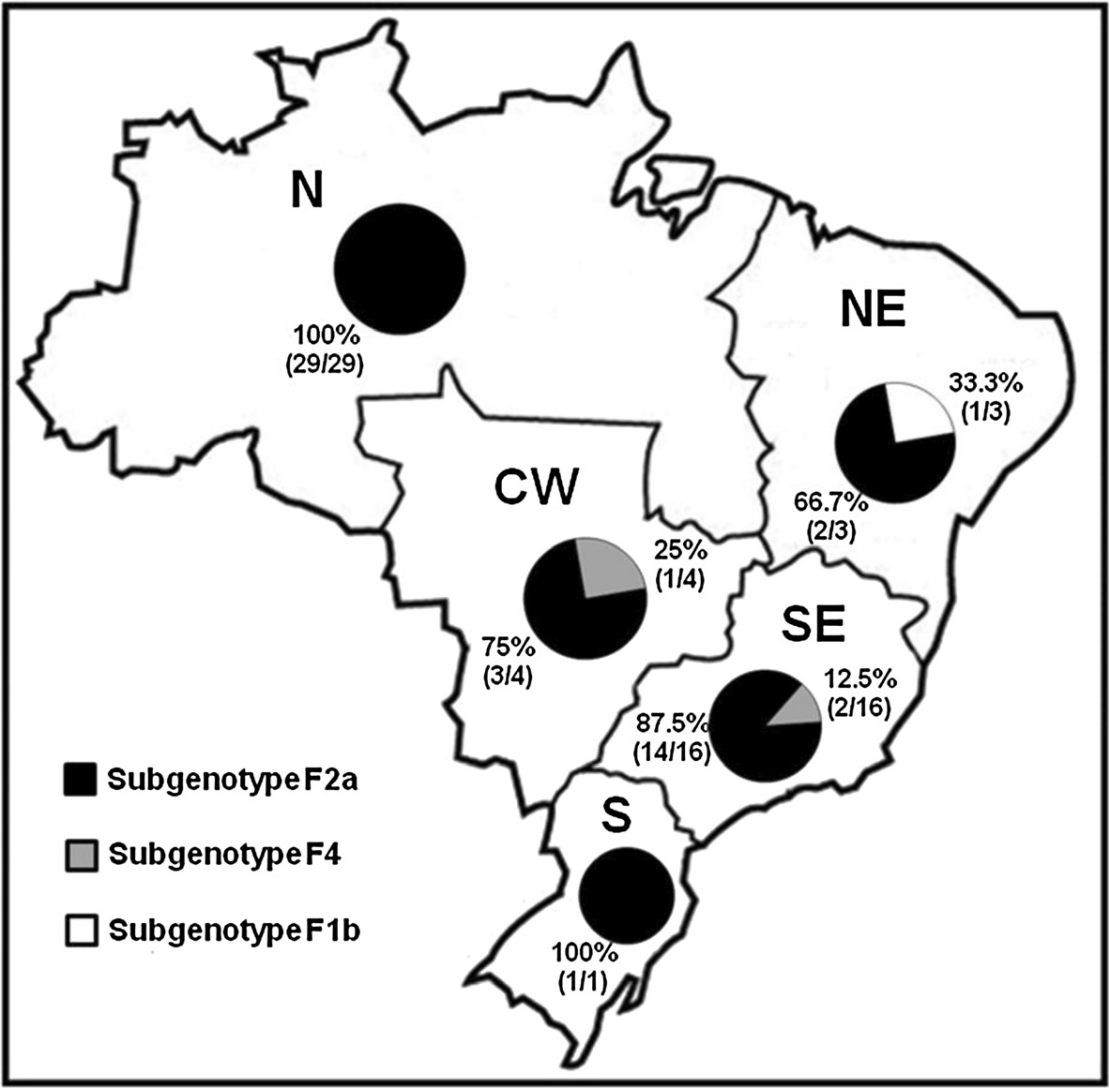
Distribution of HBV/F subgenotypes in the Brazilian geographic regions (N - north; NE - northeast; CW - central west; SE - southeast; S - south), based on 53 HBV/F S-gene sequences from Brazil available in GenBank.

Concerning the geographic distributions of these subgenotypes, HBV/F2a was found in all five Brazilian geographic regions and corresponded to 100% (29/29) of the sequences from the north, 100% (1/1) from the south, 87.5% (14/16) from the southeast, 75% (3/4) from the central west, and 66.7% (2/3) from the northeast. HBV/F1b was found in 33.3% (1/3) of the sequences from the northeast, whereas HBV/F4 was found in 25% (1/4) of the sequences from the central west and 12.5% (2/16) of the sequences from the southeast (Figure 2).

Thus, we obtained 12 full-length HBV sequences from different Brazilian geographic regions representing the three HBV/F subgenotypes circulating in Brazil: HBV/F2a (north, n=1; northeast, n=3; central west, n=1; southeast, n=4), HBV/F1b (northeast, n=1; southeast, n=1) and HBV/F4 (southeast, n=1) (Additional file 1: Table S1). Interestingly, one HBV/F1b complete genome sequence was found in the southeast, where this subgenotype had not been previously detected. These sequences were used in our Bayesian coalescent analysis.

### Bayesian analyses of HBV/F complete genome sequences

**Figure 3:**
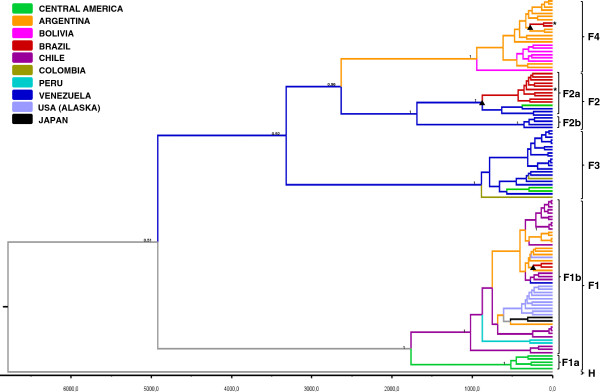
**Time-scaled Bayesian Maximum Clade Credibility tree of HBV/F full length genome sequences calibrated with the substitution rate of 1.0 × 10**^**-5 **^**s/s/y.** The branches are colored according to the most probable location of their parental node (see the color legend in the figure). The numbers on the internal nodes represent posterior probabilities (pp), and the branch lengths of the tree correspond to length of time (see the time scale bar at the bottom of the tree). The symbol “▲” indicates the nodes corresponding to the posterior root state probability (*PRSP*) values reported for HBV/F1b-BR, HBV/F2a-BR and HBV/F4-BR (0.91, 0.75, and 0.99, respectively). The two Brazilian HBV/F complete genome sequences previously available in GenBank are identified with *.

To examine the time and starting point of diversification of HBV/F subgenotypes in Brazil, we conducted Bayesian Markov chain Monte Carlo (MCMC) analyses on 117 complete genome sequences (12 from our study and 105 from GenBank). We estimated the time of the most recent common ancestor (tMRCA) of all internal nodes of the maximum clade credibility (MCC) tree under a relaxed molecular clock model. Since there is not a consensus on the HBV substitution rate, we used three previously published substitution rates: (i) 7.72 × 10^-4^ s/s/y obtained from Bayesian approaches using complete genome sequences [6], (ii) an “intermediate” rate in the range of previous estimates of 1.0 × 10^-5^ s/s/y that was chosen by Torres and co-authors to test one of the possible scenarios for the evolutionary history of HBV/F [26] and (iii) a slower nucleotide substitution rate of 2.2 × 10^-6^ s/s/y recently estimated using deep calibration ages [8]. Because substitution rates ranged among 10^-4^ to 10^-6^, a great variation of tMRCAs was found for HBV/F and its subgenotypes (Table 1). However, an older median ancestral age for the Brazilian HBV/F2a (HBV/F2a-BR) subgenotype compared to HBV/F1b-BR and HBV/F4-BR could be estimated (Table 1). Figure 3 shows the time-scaled Bayesian MCC tree, in which the sequences clustered into highly supported monophyletic clades corresponding to the four HBV/F subgenotypes (HBV/F1-F4). Our analyses indicated that most of the posterior root state probability (*PRSP*) mass of HBV/F1b-BR and HBV/F4-BR was placed in Argentina (*PRSP* = 0.91 and *PRSP* = 0.99, respectively), while HBV/F2a-BR root location was most likely Venezuela (*PRSP* = 0.75) (Figure 3). Therefore, these data suggest that Argentina and Venezuela were the most probable locations from where HBV/F1b and HBV/F4, and HBV/F2a, respectively, were introduced into Brazil.

**Table 1:** Estimated tMRCAs for HBV/F complete genome sequences (GenBank accession numbers available in Supplementary file 2

**Group**	**tMRCA (95% HPD) years**		
	7.72 x 10^-4^	1.0 x 10^-5^	2.2 x 10^-6^
F/H	85 (37 – 178)	6,785 (3,468 – 13,857)	31,303 (14,989 – 58,828)
F	62 (33 – 101)	4,919 (2,984 – 8,978)	22,522 (13,401 – 35,422)
F1	21 (12 – 38)	1,764 (964 – 3,081)	7,753 (4,122 – 13,605)
F1a	6 (3 – 12)	520 (269 – 936)	2,358 (1,229 – 4,077)
F1b	13 (7 – 19)	1,020 (627 – 1,564)	4,483 (2,901 – 6,899)
F2	22 (12 – 35)	1,687 (870 – 2,865)	7,235 (4,103 – 11,433)
F2a	11 (6 – 18)	876 (506 – 1,433)	3,807 (2,221 – 5,973)
F2b	4 (2 – 8)	353 (204 – 608)	1,532 (850 – 2,626)
F3	11 (6 – 19)	884 (480 – 1,554)	3,961 (2,130 – 6,777)
F4	12 (7 – 21)	941 (503 – 1,643)	4,065 (2,347 – 6,824)
F1b-BR	2 (1 – 3)	137 (40 – 259)	587 (191 – 1,118)
F2a-BR	5 (3 – 7)	425 (278 – 668)	1,890 (1,201 – 3,006)
F4-BR	1 (0 – 3)	108 (14 – 263)	473 (62 – 1,143)

tMRCAs were calculated using three different substitution rates (7.72 x 10^-4^[6], 1.0 x 10^-5^[26] and 2.2 x 10^-6^[8] substitutions per site per year).

## Discussion

HBV/F is thought to be the original genotype of the aboriginal populations of the Americas, since it has been found in high frequencies in several countries of South and Central America [19,24,30-32,39], as well as in native Alaskan populations [21,22]. HBV/H is also considered an Amerindian genotype, as it is found in Central America, primarily in Mexico and Nicaragua [9,40]. Since HBV/F and HBV/H are closely related, it has been suggested that these genotypes likely split off from each other within the New World, via division of an ancestral HBV strain carried by the first settlers that entered the continent across the Bering Strait around 15,000 years ago [9,41]. It has been estimated that the origin of HBV/F and its subgenotypes precedes the European discovery of the Americas by around 500 years ago [8,26]. Remarkably, over the first century and a half after the conquest of the Americas, the native population plummeted by an estimated 80% (from around 50 million in 1492 to 8 million in 1650) [42]. This considerable reduction in the population size may have led to the disappearance of some HBV/F strains that were previously endemic in the continent. However, the increase of the Latin American population from the early 1800’s may have favoured transmission of HBV/F in a highly endemic manner, fixing the HBV/F1-F4 subgenotypes in the continent [26].

Unlike the other Latin American countries, where HBV/F prevails [20,30-32], Brazil has a low countrywide prevalence of HBV/F [23]. Even in the northern region of Brazil (which includes the Amazon basin, where the native Amerindian population predominates more than in other regions of the country), low frequencies of HBV/F have been detected [23,43-46]. Instead, a predominance of HBV/A, mainly HBV/A1, which was introduced to the Americas probably during the slave trade [28], has been observed. This may indicate that there has been a change in the native genotypic profile of the region, possibly due to the migratory influx that has occurred in western Amazonia since the late 19^th^ and early 20^th^ centuries, and the gradual reduction of indigenous communities over time [47]. Although the low prevalence of HBV/F countrywide, our survey of HBV/F S-gene sequences from Brazil available at GenBank showed that HBV/F is found in all Brazilian geographic regions, with most of the sequences (54.7%) isolated in the north region, and there is circulation of three subgenotypes (HBV/F2a, HBV/F4 and HBV/F1b).

Because there is no consensus about HBV evolutionary rate, with most estimates ranging between 10^-4^ to 10^−6^ s/s/y, we decided to estimate the tMRCAs of the HBV/F subgenotypes circulating in Brazil using three previously published substitution rates, 7.72 × 10^-4^[6], 1.0 × 10^-5^[26] and 2.2 × 10^-6^ s/s/y [8]. Although the substitution rate of 2.2 × 10^-6^ s/s/y was not obtained for the complete genome, most tMRCA estimations from our study and Paraskevis et al. [8] displayed an overlap of 95% HPD intervals. Among the HBV/F subgenotypes circulating in Brazil, our analysis suggested an older ancestral age for HBV/F2a-BR compared to HBV/F1b-BR and HBV/F4-BR. The median tMRCAs of HBV/F1b-BR and HBV/F4-BR were very similar, which may suggest that they appeared almost simultaneously. Inversely, HBV/F2a has a lower tMRCA than HBV/F1b and HBV/F4 when all HBV/F sequences were compared (Table 1). This fact indicates that HBV/F2a probably emerged in the American continent at a later time than the other two clades. Despite this, HBV/F2a probably arrived and started to spread in Brazil at an earlier time than HBV/F1b and HBV/F4. Since Brazil was not pointed as the epicenter of dissemination of any HBV/F clade, the time at which each clade started to spread in South America is different from the time at which each HBV/F clade started to spread in Brazil.

The tMRCA estimations using the substitution rates of 1.0 × 10^-5^ and 2.2 × 10^-6^ s/s/y (but not 7.72 × 10^-4^ s/s/y), provided epidemiologically realistic scenarios for the evolutionary history of HBV/F and its subgenotypes. Both evolutionary rates supported the hypothesis that the starting point of diversification of the strains ancestral to HBV/F2a-BR in Brazil would have occurred in the pre-Columbian Americas. However, some cautions are necessary when interpreting these results, since the number of HBV/F2a-BR sequences is greater than HBV/F1b-BR and HBV/F4-BR sequences and this may introduce a bias in tMRCA estimates. Noteworthy, HBV/F2a has been found in all geographic regions of Brazil, and is much more prevalent (92.5%) than HBV/F4 (5.7%) and HBV/F1b (1.9%). In addition, all HBV/F sequences from the Brazilian Amazon region correspond to HBV/F2a, including five sequences (EF690487, EF690488, EF690489, EF690490, and EF690491) from the native Amerindian Apurinã Tribe, thus corroborating the idea that HBV/F2a is the oldest HBV/F subgenotype in Brazil and may represent the original native HBV of the Brazilian population.

HBV/F subgenotypes have a distinct geographical distribution in the American continent. HBV/F1 has been further subdivided into two sub-clusters, HBV/F1a and HBV/F1b [39,48]. HBV/F1a remained restricted to Central America, whereas HBV/F1b had a more complex distribution, being found mainly in South America (Argentina, Brazil, Chile, Peru, and Venezuela). Curiously, HBV/F1b is also found in the native populations of Alaska. In our analysis, the Alaskan sequences grouped far from the HBV/F1 root, suggesting a relatively recent migration. This notion is supported by a paper that also suggested a relatively recent introduction of this genotype in Alaska [21]. Likewise, HBV/F2 separated into two sub-clusters, HBV/F2a and HBV/F2b [20]. HBV/F2b remained restricted to Venezuela, whereas HBV/F2a is found almost exclusively in Brazil and Venezuela, with the exception of one HBV/F2a sequence (GenBank accession number AY090455) isolated in Nicaragua. HBV/F3 is found in Central America, Colombia and Venezuela, while HBV/F4 in Argentina, Bolivia and Brazil. Our data suggest a plausible route of introduction of HBV/F subgenotypes in Brazil. The phylogeographic patterns indicated that Venezuela was the most probable origin for HBV/F2a that has subsequently spread countrywide, indicating a north-to-south net viral flow in Brazil. On the other hand, although the limited number of Brazilian HBV/F1b and HBV/F4 sequences, these strains appeared to have spread from the south to the north, being Argentina the most probable epicenter of introduction of these subgenotypes in Brazil.

## Conclusions

We herein employed the Bayesian coalescent and phylogeographic framework to investigate the evolutionary history of HBV/F and the complex factors underlying its origin in Brazil. Our study suggests a plausible route of introduction of HBV/F subgenotypes in Brazil and provides full-length genomic sequences of HBV/F isolates from different Brazilian geographic regions, which will increase the contribution of Brazilian isolates to further phylogenetic studies of HBV/F.

## Methods

### DNA extraction and molecular assays

HBV-DNA were extracted from a total of 29 HBeAg positive serum samples taken from different geographic regions of Brazil, previously characterized as having HBV/F strains. However, the amplification and sequencing of the complete genome were successfully performed in 12 samples: southeast (n=6), northeast (n=4), central west (n=1), and north (n=1) (Additional file 1: Table S1). The Ethics Committee of Oswaldo Cruz Institute reviewed and approved the research protocol. All participating individuals gave written informed consent.

HBV DNA was extracted from 0.2 mL of serum using a High Pure Viral Nucleic Acid kit (Roche Diagnostics, Germany) according to the manufacturer’s instructions. The full-length HBV genome was amplified as previously described [49] and purified using the Wizard SV Gel and PCR Clean-Up System (Promega, WI, USA). HBV nucleotide sequences were determined using a BigDye Terminator kit (Applied Biosystems, CA, USA) and the previously described primers [50]. Sequencing reactions were analyzed on an ABI3730 automated sequencer (Applied Biosystems). The nucleotide sequences obtained during this study were deposited in the GenBank database under accession numbers KC494394-KC494405.

### HBV/F S-gene sequence dataset from Brazil

All HBV/F S-gene sequences from Brazil available in GenBank by October 2012 (n=53) were selected for phylogenetic analysis. Reference sequences for HBV genotypes A-J were also included in the analysis. The phylogenetic relationships among the S-gene sequences were evaluated with the neighbor-joining method (1000 bootstrap replicates) using MEGA version 5.1 [51].

### Bayesian phylogenetic and phylogeographic analyses

The 12 complete HBV/F genome sequences generated in this study were combined with 105 full-length HBV/F sequences from North, Central and South America and Japan (GenBank accession numbers available in Additional file 2: Table S2). All selected sequences had country data available and were classified as non-recombinant. The sampling locations included Argentina (n=24), Bolivia (n=7), Brazil (n=2), Chile (n=21), Colombia (n=2), Japan (n=2), Peru (n=2), USA (n=11) and Venezuela (n=26). The sequences from Costa Rica (n=2), El Salvador (n=2), Nicaragua (n=2), and Panama (n=2) were grouped as representing Central America. A reference sequence from HBV/H was also included in the analysis.

The tMRCA and the spatial diffusion for the full-length sequences of HBV/F and its subgenotypes were jointly estimated using the Bayesian MCMC statistical framework implemented in the BEAST v1.7.4 package [52,53]. A matrix of geographic locations was constructed based on the sampling location for each sequence. A full model was used in which all possible reversible exchange rates between locations were equally likely (flat prior) [54]. Analyses were carried out with a Bayesian Skyline coalescent tree [55] under the model of nucleotide substitution TVM + Γ + I, which was selected as the best-fit model by the jModeltest program [56]. We used a relaxed (uncorrelated Lognormal) molecular clock model [57] that was chosen over a strict molecular clock by calculating the Bayes Factor from the posterior output of each model using TRACER v1.4 [58]. The time-scale of the Bayesian tree was calibrated using three previously published substitution rates, 7.72 × 10^-4^[6], 1.0 × 10^-5^[26] and 2.2 × 10^-6^ s/s/y [8]. MCMC analysis was run for 5 × 10^7^ generations to achieve the convergence of parameters, which was assessed after a 10% burn-in and calculation of the Effective Sample Size (ESS) using TRACER v1.4. All parameter estimates showed ESS values >200, and their uncertainty was reflected in the 95% Highest Posterior Density (HPD) intervals. The MCC tree was visualized using the FigTree v.1.3.1 program after the posterior tree distribution was summarized using the TreeAnnotator v.1.7.4 program.

## Abbreviations

HBV: Hepatitis B virus; s/s/y: Substitutions per site per year; HDV: Hepatitis delta virus; MCMC: Markov chain Monte Carlo; tMRCA: Most recent common ancestor; MCC: Maximum clade credibility; PRSP: Posterior root state probability; HPD: Highest posterior density; pp: Posterior probabilities.

## Competing interests

The authors declare no competing interests.

## Authors’ contributions

FCAM carried out the molecular biology experiments, constructed the HBV sequence dataset and helped to draft the manuscript. OCA conducted the sequence alignment. BVL participated in the sequencing process. ARMT, MTBM participated in the design of the study. SAG participated in the design of the study and helped to draft the manuscript. GB carried out the Bayesian phylogenetic and phylogeographic analyses and helped to draft the manuscript. NMA conceived the study, participated in its design and coordination, and drafted the manuscript. All authors read and approved the final manuscript.

## Supplementary Material

Additional file 1: Table S1 Data of the sequences obtained in this study. Click here for file

Additional file 2: Table S2 GenBank accession numbers of the 105 full-length HBV/F sequences used in the Bayesian phylogenetic and phylogeographic analyses. Click here for file
