# Supplementary material for: Phylogeography and evolutionary history of hepatitis B virus genotype F in Brazil
Source: Virol J. 2013 Jul 16;10:236. doi: 10.1186/1743-422X-10-236 (PMC3751091; doi:10.1186/1743-422X-10-236)
Supplement: Additional file 1: Table S1 — Data of the sequences obtained in this study. [file 1743-422X-10-236-S1.doc]

**Supplementary file 1:** Data of the sequences obtained in this study

| **GenBank accession**  **number** | **Isolate** | **Subgenotype** | **HBeAg**  **status** | **Brazilian State**  **(geographic region)** | **Origin of the sample** |
| --- | --- | --- | --- | --- | --- |
| KC494405 | BR1-NORTHEAST | F2a | Pos | Pernambuco (northeast) | Blood donor |
| KC494404 | BR2-NORTHEAST | F1b | Pos | Pernambuco (northeast) | Blood donor |
| KC494403 | BR3-NORTHEAST | F2a | Pos | Pernambuco (northeast) | Blood donor |
| KC494402 | BR4-NORTHEAST | F2a | Pos | Pernambuco (northeast) | Blood donor |
| KC494401 | BR5-SOUTHEAST | F2a | Pos | Rio de Janeiro (southeast) | Chronic infection |
| KC494400 | BR6-SOUTHEAST | F1b | Pos | Rio de Janeiro (southeast) | Chronic infection |
| KC494399 | BR7-CENTRAL WEST | F2a | Pos | Mato Grosso do Sul (central west) | Chronic infection |
| KC494398 | BR8-SOUTHEAST | F4 | Pos | Rio de Janeiro (southeast) | Chronic infection |
| KC494397 | BR9-SOUTHEAST | F2a | Pos | Rio de Janeiro (southeast) | Chronic infection |
| KC494396 | BR10-SOUTHEAST | F2a | Pos | Rio de Janeiro (southeast) | Chronic infection |
| KC494395 | BR11-SOUTHEAST | F2a | Pos | Rio de Janeiro (southeast) | Blood donor |
| KC494394 | BR12-NORTH | F2a | Pos | Amapá (north) | Blood donor |
