# Supplementary material for: Phylogeography and evolutionary history of hepatitis B virus genotype F in Brazil
Source: Virol J. 2013 Jul 16;10:236. doi: 10.1186/1743-422X-10-236 (PMC3751091; doi:10.1186/1743-422X-10-236)
Supplement: Additional file 2: Table S2 — GenBank accession numbers of the 105 full-length HBV/F sequences used in the Bayesian phylogenetic and phylogeographic analyses. [file 1743-422X-10-236-S2.doc]

**Supplementary file 2:** GenBank accession numbers of the 105 full-length HBV/F sequences used in the Bayesian phylogenetic and phylogeographic analyses.

| AB036905 | AB116551 | AY090461 | DQ899146 | HE981181 | HM590473 |
| --- | --- | --- | --- | --- | --- |
| AB036907 | AB116552 | AY179734 | DQ899147 | HM585186 | HM590474 |
| AB036908 | AB116654 | AY179735 | DQ899149 | HM585187 | HM622135 |
| AB036909 | AB166850 | AY311369 | DQ899150 | HM585188 | HM627320 |
| AB036910 | AB365446 | AY311370 | EU366116 | HM585189 | JN792913 |
| AB036911 | AB365447 | DQ776247 | EU366118 | HM585190 | JN792914 |
| AB036912 | AB365448 | DQ823086 | EU366132 | HM585191 | JN792915 |
| AB036913 | AB365449 | DQ823087 | EU366133 | HM585192 | JN792916 |
| AB036914 | AB365450 | DQ823088 | EU670261 | HM585193 | JN792917 |
| AB036915 | AB365453 | DQ823089 | EU670262 | HM585194 | JN792918 |
| AB036916 | AF223962 | DQ823090 | FJ589065 | HM585195 | JN792919 |
| AB036917 | AF223963 | DQ823091 | FJ589066 | HM585196 | JN792920 |
| AB036919 | AF223964 | DQ823094 | FJ589067 | HM585197 | JN792921 |
| AB036920 | AF223965 | DQ823095 | FJ657519 | HM585198 | JN792922 |
| AB064316 | AY090455 | DQ899142 | FJ657522 | HM585199 | X69798 |
| AB086397 | AY090456 | DQ899143 | FJ657525 | HM585200 |  |
| AB116549 | AY090458 | DQ899144 | FJ657528 | HM590471 |  |
| AB116550 | AY090459 | DQ899145 | FJ657529 | HM590472 |  |
